# Bioinformatic Analysis Identifies Potential Key Genes in the Pathogenesis of Turner Syndrome

**DOI:** 10.3389/fendo.2020.00104

**Published:** 2020-03-06

**Authors:** Hao Wang, Hui Zhu, Wenjiao Zhu, Yue Xu, Nan Wang, Bing Han, Huaidong Song, Jie Qiao

**Affiliations:** ^1^Department of Endocrinology, Shanghai Ninth People's Hospital, Shanghai Jiao Tong University School of Medicine, Shanghai, China; ^2^Research Centre for Clinical Medicine, Shanghai Ninth People's Hospital, Shanghai Jiao Tong University School of Medicine, Shanghai, China

**Keywords:** turner syndrome, microarray expression profiling dataset, differentially expressed genes, protein-protein interaction network, tissue-specific gene expression

## Abstract

**Background:** Turner syndrome (TS) is a sex chromosome aneuploidy with a variable spectrum of symptoms including short stature, ovarian failure and skeletal abnormalities. The etiology of TS is complex, and the mechanisms driving its pathogenesis remain unclear.

**Methods:** In our study, we used the online Gene Expression Omnibus (GEO) microarray expression profiling dataset GSE46687 to identify differentially expressed genes (DEGs) between monosomy X TS patients and normal female individuals. The relevant data on 26 subjects with TS (45,XO) and 10 subjects with the normal karyotype (46,XX) was investigated. Then, tissue-specific gene expression, functional enrichment, and protein-protein interaction (PPI) network analyses were performed, and the key modules were identified.

**Results:** In total, 25 upregulated and 60 downregulated genes were identified in the differential expression analysis. The tissue-specific gene expression analysis of the DEGs revealed that the system with the most highly enriched tissue-specific gene expression was the hematologic/immune system, followed by the skin/skeletal muscle and neurologic systems. The PPI network analysis, construction of key modules and manual screening of tissue-specific gene expression resulted in the identification of the following five genes of interest: *CD99, CSF2RA, MYL9, MYLPF*, and *IGFBP2*. *CD99* and *CSF2RA* are involved in the hematologic/immune system, *MYL9* and *MYLPF* are related to the circulatory system, and *IGFBP2* is related to skeletal abnormalities. In addition, several genes of interest with possible roles in the pathogenesis of TS were identified as being associated with the hematologic/immune system or metabolism.

**Conclusion:** This discovery-driven analysis may be a useful method for elucidating novel mechanisms underlying TS. However, more experiments are needed to further explore the relationships between these genes and TS in the future.

## Introduction

Turner syndrome (TS) is a common genetic condition caused by abnormal sex chromosomes that affects 1 in 2500 female live births (1). Since initially described by Henry Turner in 1938, TS was gradually recognized as a syndrome characterized by the complete absence or partial loss of an X chromosome in phenotypic females. The clinical signs of TS include short stature, gonadal dysgenesis, lymphedema webbed neck and other more than 400 types of dysmorphic features. In addition, cardiovascular disease are more prevalent in women with TS, including congenital cardiac abnormalities, aortic dilation and dissection, hypertension and ischemic heart disease (2). In addition, ~30% of individuals with TS have bicuspid aortic valve (BAV) (3). Typically, nearly half the patients with TS have a 45,X karyotype, 20–30% of patients with TS present with mosaicism (45,XO/46,XX), and the remainder have X chromosome structural abnormalities (4). Females with TS present with highly variable clinical features, which are caused by the haploinsufficiency of genes involved in multiple systems.

TS is a multiple-system disease, and the etiology is complex. However, the mechanisms underlying the pathogenesis of TS remain unclear. Previous studies indicated that haploinsufficiency of the *short stature homeobox* (*SHOX*) gene leads to occurrence of short stature and many specific skeletal anomalies in TS individuals (5). However, as a mainly developmental disorder, the pathogenesis of congenital heart defects in TS stills unclear. In previous research, mutations in *NKX2.5* (6), *GATA5* (7), and *NOTCH1* (8) have been identified as the causative factor in non-syndromic patients with inherited BAV. Moreover, chromosome structural variants and potential pathogenic genes such as *TIMP3* and *TIMP1* may be associated with TS patients with congenital cardiac abnormalities (3, 9). In addition, sex chromosome imbalance and dysregulation of certain genes on the X chromosome (such as *FMR1, PDIAPH2*, and *BMP15*, etc.) may result in accelerated oocyte atresia, leading to gonadal dysgenesis later in life (10, 11). And haploinsufficiency of a lymphatic gene is related to the development of lymphedema and webbed neck (12). Recently, haploinsufficiency of immune-associated genes on the X chromosome was reported to result in the development of autoimmune diseases, including autoimmune thyroiditis, type 1 diabetes and autoimmune enteritis (13).

Altered autosomal gene expression as well as chrX gene expression has been observed in females with TS (45,X monosomy) in different samples as human fibroblast cell line, peripheral blood mononuclear cells, as well as in the induced pluripotent human cell lines, with inconsistent results. However, further data analysis and data mining are still absent, especially the derivation of X chromosome of the patients who inherited the monosomy X chromosome from mother or father (TS with Xm and Xp). Therefore, we used the statistical analysis and some data mining techniques to reveal patterns of genes responsible for TS. Here, we used the peripheral blood mononuclear cell (PBMC) microarray dataset GSE46687 created by Bondy et al. to perform a genome-wide gene expression analysis to investigate the differentially expressed genes (DEGs) between monosomy X TS patients and normal female 46,XX individuals to understand postnatal differences. Our results will contribute to our understanding of the genetic etiology of TS and provide new insights into the clinical diagnosis and treatment of TS.

## Materials and Methods

### Microarray Data

The microarray expression profiling dataset GSE46687, deposited by Bondy et al., was downloaded from the Gene Expression Omnibus (GEO, https://www.ncbi.nlm.nih.gov/geo/). The dataset was based on the GPL570 Affymetrix Human Genome U133 Plus 2.0 Array platform. The experiment contained 36 samples consisting of 16 subjects with TS who were identified as having a maternally inherited X chromosome (45,Xm), 10 subjects with TS who were identified as having a paternally inherited X chromosome (45,Xp) and 10 subjects with the normal female karyotype (46,XX). Since it was public dataset, the information of age and health condition as well as the usage of the medication of the individuals was unavailable, which appears to be a potential limitation. The annotation file for GPL570 was also downloaded from the GEO.

### Differential Expression Analysis

Differential expression analysis was performed using the online analysis tool GEO2R; the expression profiles of monosomy X TS patients and normal 46,XX females were compared to identify the DEGs. *P*-values and adjusted *P*-values were calculated using *t*-tests. Genes from each sample with the following criteria were retained: (1) a |log2 (fold-change)| >1 and (2) an adjusted *P* < 0.05. We selected the most significant genes when the DEGs were duplicated. We divided the TS patients into two groups depending on the parental origin of the existing X chromosome. Analyses were performed independently for the 45,Xm and 45,Xp TS samples, and the DEGs were determined by the intersection of the two datasets. A Venn diagram of DEGs was drawn using the online tool Venny 2.1 (http://bioinfogp.cnb.csic.es/tools/venny/index.html), and the heatmap for the DEGs was created using Heml software.

### Tissue-Specific Gene Expression Analysis

We used the online resource BioGPS (http://biogps.org) to analyze the tissue-specific expression of the DEGs. Transcripts mapped to a single tissue with the following criteria were identified as highly tissue specific: (1) the tissue-specific expression level was >10 times the median, and (2) the second highest expression level was less than one-third as high as the highest level (14).

### Functional Enrichment Analysis of DEGs

We used DAVID 6.8 (https://david.ncifcrf.gov/tools.jsp) to perform the functional enrichment analysis of DEGs; this analysis included the functional categories, Gene Ontology (GO) terms and Kyoto Encyclopedia of Genes and Genomes (KEGG) pathways. The GO analysis included 3 categories, namely, biological process (BP), cellular component (CC) and molecular function (MF), which were used to predict protein functions (15). KEGG pathway analysis was used to assign sets of DEGs to specific pathways to enable the construction of the molecular interaction, reaction and relationship networks (16). The functional categories included COG_ONTOLOGY, UP_KEYWORDS and UP_SEQ_FEATURE. Benjamini-adjusted *P* < 0.05 and an enriched gene count >5 were chosen as the criteria for significance.

### Protein-Protein Interaction (PPI) Network Analysis

The PPI network analysis was conducted using STRING (https://string-db.org/), which is an online database of known and predicted protein-protein interactions. These interactions include physical and functional associations, and the data are mainly derived from computational predictions, high-throughput experiments, automated text mining and co-expression networks. We mapped the DEGs onto the PPI network and set an interaction score of >0.4 as the threshold value. In addition, Cytoscape v3.6.0 software was used to visualize and construct the PPI network. Nodes with the greatest numbers of interactions with neighboring nodes were considered hub nodes.

To identify the key PPI network modules, the app ClusterOne from the Cytoscape software suite was used to perform the gene network clustering analysis. A *P* < 0.05 was set as the significance threshold for identifying key modules.

## Results

### Differentially Expressed Genes

**Figure 1:**
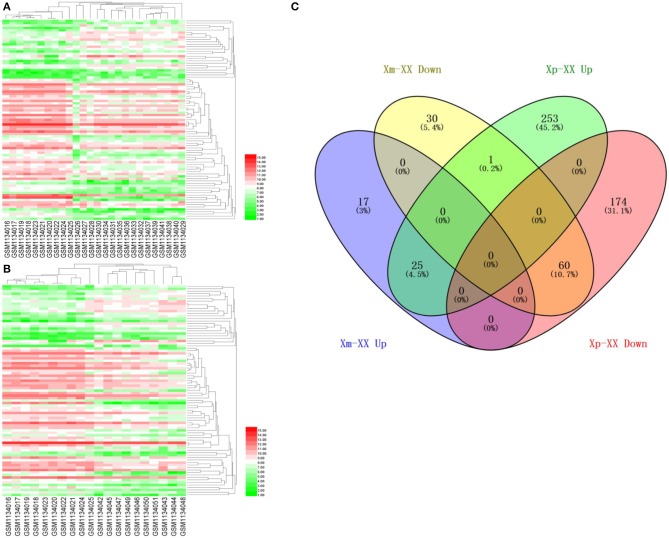
A heatmap of 85 differentially expressed genes between monosomy X TS patients and 46,XX normal individuals. **(A)** Xm TS patients and normal individuals. **(B)** Xp TS patients and normal individuals. Red represents upregulated genes, and green represents downregulated genes. **(C)** Venn diagram of differentially expressed genes between monosomy X TS patients and 46,XX normal individuals. Up represents upregulated genes, and down represents downregulated genes.

We downloaded the microarray expression dataset GSE46687 from the GEO database and analyzed the DEGs between monosomy X TS patients and normal female 46,XX individuals using the online analysis tool GEO2R. In total, 42 upregulated and 91 downregulated genes were identified between Xm TS patients and normal individuals. In addition, 279 upregulated and 234 downregulated genes were identified between Xp TS patients and normal individuals (Supplemental Table 1). We identified the intersection of these two datasets and obtained a total of 25 upregulated and 60 downregulated genes. As shown in Table 1, 15 (17.6%, one upregulated and 14 downregulated) of the 85 DEGs were on the X chromosome. Most of these genes are involved in basic cellular activities, such as the structural maintenance of chromosomes and the mediation of transcription. Three of the downregulated genes (*AP1S2, CSF2RA*, and *CD99*) on the X chromosome were related to the immune system. The *X inactive specific transcript* (*XIST*) gene was downregulated, and the adjusted *P*-value was highly significant (*P* < 0.0001). The Venn diagram and heatmap for the DEGs are presented in Figure 1. As shown in Figures 1A,B, 25 upregulated and 60 downregulated genes were identified through the comparison of TS patients and normal individuals. LMF1, a protein-coding gene involved in the maturation and transport of lipoprotein lipase, was upregulated in Xp TS patients, whereas it was downregulated in Xm TS patients (Figure 1C).

**Table 1:** Differentially expressed genes of Turner Syndrome.

**Gene symbol**	**Adjusted** ***P*****-value**		**Fold change**		**Gene title**	**Location**
	**Xm-XX**	**Xp-XX**	**Xm-XX**	**Xp-XX**		
**Up regulated genes**						
ACVR2B	1.79E-02	8.62E-03	4.659	4.852	Activin A receptor type 2B	Chromosome 3
AGER	2.44E-02	1.27E-02	3.434	3.729	Advanced glycosylation end-product specific receptor	Chromosome 6
ANK1	2.12E-02	3.55E-03	5.540	2.630	Ankyrin 1	Chromosome 8
B3GAT1	4.31E-02	3.80E-02	4.993	5.448	Beta-1,3-glucuronyltransferase 1	Chromosome 11
BAZ2A	1.73E-02	1.35E-02	2.639	2.662	Bromodomain adjacent to zinc finger domain 2A	Chromosome 12
BCL11B	2.50E-02	1.27E-02	3.074	3.550	B-cell CLL/lymphoma 11B	Chromosome 14
C11orf53	3.41E-02	1.31E-02	2.969	2.701	Chromosome 11 open reading frame 53	Chromosome 11
CEMP1	3.83E-02	4.53E-03	2.114	2.665	Cementum protein 1	Chromosome 16
CYBA	4.90E-02	4.44E-03	2.428	2.951	Cytochrome b-245 alpha chain	Chromosome 16
DGKK	9.00E-04	2.65E-02	6.916	4.562	Diacylglycerol kinase kappa	Chromosome X
FAM229B	4.53E-02	2.20E-02	2.969	2.821	Family with sequence similarity 229 member B	Chromosome 6
HDAC5	2.13E-02	2.05E-05	2.173	2.459	Histone deacetylase 5	Chromosome 17
IGFBP2	4.73E-02	1.74E-02	4.347	3.908	Insulin like growth factor binding protein 2	Chromosome 2
KDM6B	7.00E-03	8.66E-03	3.387	3.502	Lysine demethylase 6B	Chromosome 17
KHSRP	3.83E-02	2.97E-02	2.378	2.391	KH-type splicing regulatory protein	Chromosome 19
MED16	4.89E-02	1.17E-03	2.042	2.478	Mediator complex subunit 16	Chromosome 19
MYL9	2.95E-02	3.45E-02	10.411	9.513	Myosin light chain 9	Chromosome 20
OVCH1-AS1	3.83E-02	6.05E-03	3.387	4.555	OVCH1 antisense RNA 1	Chromosome 12
PERM1	3.12E-02	3.24E-02	2.713	2.050	PPARGC1 and ESRR induced regulator, muscle 1	Chromosome 1
PSMD5-AS1	4.12E-02	2.10E-02	2.908	2.748	PSMD5 antisense RNA 1 (head to head)	Chromosome 9
SAFB2	4.89E-02	5.15E-03	2.532	3.022	Scaffold attachment factor B2	Chromosome 19
SRCAP	1.37E-02	8.18E-05	4.563	6.930	Snf2-related CREBBP activator protein	Chromosome 16
STK11	6.00E-04	2.48E-03	6.063	6.820	Serine/threonine kinase 11	Chromosome 19
UBE2O	9.00E-03	4.44E-03	2.549	2.801	Ubiquitin conjugating enzyme E2 O	Chromosome 17
ZMIZ2	3.30E-02	2.81E-02	2.621	2.793	Zinc finger MIZ-type containing 2	Chromosome 7
**Down regulated genes**						
AFF3	2.54E-02	4.28E-02	0.1934	0.3026	AF4/FMR2 family member 3	Chromosome 2
AGFG1	3.41E-02	3.37E-03	0.4033	0.4174	ArfGAP with FG repeats 1	Chromosome 2
AHNAK	6.80E-03	2.74E-02	0.2132	0.2273	AHNAK nucleoprotein	Chromosome 11
AP1S2^*^	2.95E-02	2.60E-03	0.4263	0.3898	Adaptor related protein complex 1 sigma 2 subunit	Chromosome X
ASMTL	3.60E-03	3.50E-04	0.4633	0.4923	Acetylserotonin O-methyltransferase-like	Chromosome X
ATRX	1.68E-02	1.32E-02	0.3950	0.4502	ATRX, chromatin remodeler	Chromosome X
BID	3.26E-02	3.38E-03	0.4796	0.4956	BH3 interacting domain death agonist	Chromosome 22
CARD16	2.03E-02	4.77E-03	0.4569	0.4879	Caspase recruitment domain family member 16	Chromosome 11
CD99^*^	2.01E-02	3.58E-03	0.4601	0.4851	CD99 molecule	Chromosome X
CDC27	6.00E-04	1.08E-04	0.3711	0.3945	Cell division cycle 27	Chromosome 17
CHD9	3.20E-03	8.23E-03	0.3078	0.3351	Chromodomain helicase DNA binding protein 9	Chromosome 16
CSF2RA^*^	8.81E-03	5.39E-04	0.3099	0.3510	Colony stimulating factor 2 receptor alpha subunit	Chromosome X
CSGALNACT2	5.94E-03	1.18E-02	0.3842	0.3309	Chondroitin sulfate N-acetylgalactosaminyltransferase 2	Chromosome 10
CXorf38	4.00E-04	3.40E-04	0.3869	0.3602	Chromosome X open reading frame 38	Chromosome X
DHRSX	2.50E-02	6.05E-03	0.2736	0.2140	Dehydrogenase/reductase X-linked	Chromosome X
DHX9	3.08E-02	2.78E-02	0.4569	0.4841	DEAH-box helicase 9	Chromosome 1
EIF1AX	3.41E-02	5.57E-03	0.4897	0.4475	Eukaryotic translation initiation factor 1A, X-linked	Chromosome X
EPRS	1.79E-03	5.92E-03	0.4005	0.3890	Glutamyl-prolyl-tRNA synthetase	Chromosome 1
EREG	8.39E-03	4.73E-03	0.2952	0.3238	Epiregulin	Chromosome 4
FCGR2C	3.23E-03	2.59E-02	0.2365	0.4009	Fc fragment of IgG receptor Iic	Chromosome 1
FKBP15	3.23E-03	6.31E-03	0.2813	0.2773	FK506 binding protein 15	Chromosome 9
FRG1JP	4.90E-02	1.61E-02	0.3345	0.3576	FSHD region gene 1 family member J, pseudogene	Chromosome 9
FXR1	3.23E-03	2.98E-03	0.3511	0.3833	FMR1 autosomal homolog 1	Chromosome 3
HADHA	2.40E-03	2.60E-03	0.4263	0.3944	Hydroxyacyl-CoA dehydrogenase/3-ketoacyl-CoA thiolase/enoyl-CoA hydratase (trifunctional protein), alpha subunit	Chromosome 2
HECTD1	7.01E-03	8.16E-03	0.4730	0.4768	HECT domain E3 ubiquitin protein ligase 1	Chromosome 14
HES1	4.11E-02	3.19E-02	0.2973	0.4613	Hes family bHLH transcription factor 1	Chromosome 3
IL1R2	3.08E-02	1.99E-02	0.1627	0.3489	Interleukin 1 receptor type 2	Chromosome 2
INO80D	2.00E-02	5.42E-03	0.2736	0.2952	INO80 complex subunit D	Chromosome 2
IQGAP1	6.35E-04	2.48E-03	0.3487	0.3747	IQ motif containing GTPase activating protein 1	Chromosome 15
JAK1	2.03E-02	1.30E-02	0.4538	0.4758	Janus kinase 1	Chromosome 1
KDM6A	1.51E-02	4.48E-03	0.3896	0.4880	Lysine demethylase 6A	Chromosome X
KIAA1033	1.50E-03	2.77E-03	0.3789	0.4213	KIAA1033	Chromosome 12
LOC100289090	6.35E-04	5.41E-03	0.3392	0.4276	Uncharacterized LOC100289090	Chromosome 15
LOC101930114	1.52E-02	9.33E-03	0.4005	0.4218	Uncharacterized LOC101930114	Chromosome 1
MAP4	1.93E-02	1.15E-02	0.2774	0.3805	Microtubule associated protein 4	Chromosome 3
MYLPF	3.41E-02	3.58E-03	0.2872	0.1968	Myosin light chain, phosphorylatable, fast skeletal muscle	Chromosome 16
NLRC4	2.48E-02	2.60E-02	0.2793	0.2312	NLR family CARD domain containing 4	Chromosome 2
NSMAF	8.98E-04	5.51E-03	0.3487	0.2810	Neutral sphingomyelinase activation associated factor	Chromosome 8
PLXNC1	1.92E-02	1.00E-02	0.3487	0.3616	Plexin C1	Chromosome 12
PTPN12	2.82E-02	3.06E-02	0.4147	0.4630	Protein tyrosine phosphatase, non-receptor type 12	Chromosome 7
RPL37A	2.50E-02	2.10E-02	0.3209	0.3612	Ribosomal protein L37a	Chromosome 2
SAMHD1	6.88E-03	6.57E-03	0.3842	0.3232	SAM and HD domain containing deoxynucleoside triphosphate triphosphohydrolase 1	Chromosome 20
SLC25A6	9.11E-04	5.97E-06	0.3711	0.3762	Solute carrier family 25 member 6	Chromosome X
SMC1A	8.81E-03	2.03E-03	0.1661	0.3258	Structural maintenance of chromosomes 1A	Chromosome X
SON	9.93E-03	6.67E-03	0.4175	0.4495	SON DNA binding protein	Chromosome 21
SSFA2	1.68E-02	9.56E-03	0.3164	0.3670	Sperm specific antigen 2	Chromosome 2
TAOK1	2.66E-02	3.55E-03	0.4965	0.4375	TAO kinase 1	Chromosome 17
TIA1	2.30E-02	8.62E-03	0.3978	0.4613	TIA1 cytotoxic granule-associated RNA binding protein	Chromosome 2
TOB1	3.41E-02	3.21E-02	0.3711	0.4056	Transducer of ERBB2, 1	Chromosome 17
TOP1	3.83E-02	4.06E-03	0.4538	0.4516	Topoisomerase (DNA) I	Chromosome 20
TPP2	4.59E-02	2.60E-03	0.4601	0.4281	Tripeptidyl peptidase 2	Chromosome 13
TSIX	2.52E-06	8.18E-05	0.0769	0.0495	TSIX transcript, XIST antisense RNA	Chromosome X
USP10	8.02E-03	2.97E-03	0.3842	0.4655	Ubiquitin specific peptidase 10	Chromosome 16
VPS13B	1.94E-02	3.46E-02	0.3816	0.3808	Vacuolar protein sorting 13 homolog B	Chromosome 8
VPS35	2.50E-02	8.24E-03	0.3392	0.3926	VPS35 retromer complex component	Chromosome 16
XIST	3.48E-14	2.64E-08	0.0042	0.0019	X inactive specific transcript (non-protein coding)	Chromosome X
ZBTB38	2.43E-03	1.34E-02	0.4538	0.4446	Zinc finger and BTB domain containing 38	Chromosome 3
ZFX	2.95E-02	5.70E-03	0.4353	0.4679	Zinc finger protein, X-linked	Chromosome X
ZG16B	1.30E-03	6.51E-03	0.2031	0.2638	Zymogen granule protein 16B	Chromosome 16
ZNF652	3.41E-02	1.99E-02	0.3439	0.3698	Zinc finger protein 652	Chromosome 17

* *Genes that were involved in the immune system and on the X chromosome*.

### Tissue-Specific Expression of Genes

We identified 23 genes that were expressed in a specific tissue or organ system using BioGPS (Supplemental Table 2). The most highly tissue-specific expression system was the hematologic/immune system (69.6%, 16/23). The neurologic and skin/skeletal muscle systems had similar levels of enrichment (8.7%, 2/23), while the respiratory, digestive and circulatory systems had the lowest enrichment levels (4.3%, 1/23) (Table 2).

**Table 2:** Tissue-specific expressed genes identified by BioGPS.

**System**	**Genes**
Hematologic/immune	ANK1, AGFG1, BID, CD99, CDC27, DHX9, EIF1AX, EPRS, EREG, FXR1, IL1R2, IQGAP1, KIAA1033, PLXNC1, SON, TOP1
Neurologic	B3GAT1, XIST
Skin/skeletal muscle	HES1, MYLPF
Respiratory	AGER
Digestive	IGFBP2
Circulatory	MYL9

### Functional and Pathway Enrichment of DEGs

**Figure 2:**
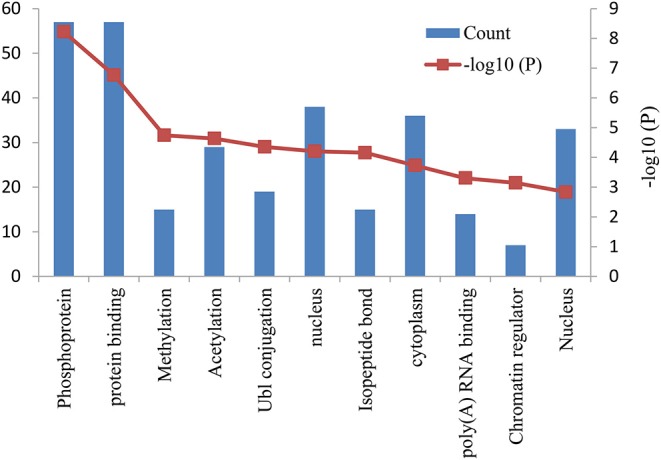
Bar graph of 11 representative enriched functional terms. The left-hand y-axis depicts the number of genes, and the right-hand y-axis depicts the –log10 (*P*-value). The x-axis lists the enriched functional terms.

The functional and pathway enrichment analyses of DEGs were performed using the DAVID 6.8 online tool. In our study, 37 enriched functional category terms, 45 enriched GO terms and 2 KEGG pathways were identified. The enriched GO terms with *P* < 0.05 are presented in Table 3; they included protein binding (*P* = 1.72 × 10^−7^) in the MF category, nucleus (*P* = 6.28 × 10^−5^) in the CC category, cytoplasm (*P* = 1.86 × 10^−4^) in the CC category and poly A RNA binding (*P* = 4.98 × 10^−4^) in the MF category. In addition, 7 enriched UP_KEYWORDS terms with *P* < 0.05 were identified, including phosphoprotein (*P* = 6.04 × 10^−9^), methylation (*P* = 1.81 × 10^−5^), acetylation (*P* = 2.33 × 10^−5^), Ubl conjugation (*P* = 4.44 × 10^−5^), isopeptide bond (*P* = 6.95 × 10^−5^), chromatin regulator (*P* = 7.22 × 10^−4^), and nucleus (*P* = 1.46 × 10^−3^). The number of genes and *P*-values of the 11 enriched functional terms are displayed in Figure 2.

**Table 3:** The enriched terms for DEGs.

**Category**	**Term**	**Description**	**Count**	**Genes**	***P*-value**
UP_KEYWORDS	/	Phosphoprotein	57	KDM6A, CXORF38, IQGAP1, ZBTB38, TOP1, NLRC4, ANK1, TPP2, ASMTL, KIAA1033, USP10, VPS13B, INO80D, AHNAK, ZFX, UBE2O, ACVR2B, KHSRP, FKBP15, SRCAP, KDM6B, BID, PLXNC1, AGFG1, STK11, SSFA2, HADHA, ZNF652, MYL9, CHD9, BCL11B, VPS35, BAZ2A, HECTD1, DHX9, TAOK1, EPRS, CD99, SAMHD1, DGKK, AFF3, MYLPF, AGER, CDC27, PTPN12, FXR1, SAFB2, HDAC5, ATRX, CYBA, B3GAT1, SON, FCGR2C, JAK1, MAP4, SMC1A, TOB1	6.04E-09
GOTERM_MF_DIRECT	GO:0005515	Protein binding	57	IQGAP1, ZBTB38, TOP1, NLRC4, AP1S2, ANK1, TPP2, ASMTL, TIA1, EIF1AX, USP10, NSMAF, AHNAK, CSF2RA, SLC25A6, HES1, UBE2O, ACVR2B, EREG, MED16, ZMIZ2, KHSRP, FKBP15, SRCAP, KDM6B, BID, IL1R2, PLXNC1, AGFG1, STK11, HADHA, ZNF652, BCL11B, VPS35, BAZ2A, HECTD1, DHX9, TAOK1, EPRS, SAMHD1, AGER, CDC27, PTPN12, FXR1, SAFB2, HDAC5, ATRX, CYBA, SON, FCGR2C, CSGALNACT2, JAK1, MAP4, RPL37A, SMC1A, IGFBP2, TOB1	1.72E-07
UP_KEYWORDS	/	Methylation	15	DHX9, KDM6A, CXORF38, STK11, SLC25A6, MYLPF, EPRS, HADHA, SAFB2, ATRX, SON, ZMIZ2, BCL11B, KHSRP, AHNAK	1.81E-05
UP_KEYWORDS	/	Acetylation	29	BID, STK11, HADHA, IQGAP1, MYL9, CHD9, TOP1, TPP2, BCL11B, TIA1, KIAA1033, USP10, BAZ2A, AHNAK, DHX9, SLC25A6, SAMHD1, EPRS, PTPN12, SAFB2, FXR1, ATRX, HDAC5, SON, KHSRP, FKBP15, MAP4, JAK1, SMC1A	2.33E-05
UP_KEYWORDS	/	Ubl conjugation	19	BID, SSFA2, SAMHD1, SAFB2, ZBTB38, HDAC5, ATRX, CHD9, TOP1, UBE2O, SON, ZMIZ2, BCL11B, EIF1AX, KHSRP, MAP4, USP10, BAZ2A, AHNAK	4.44E-05
GOTERM_CC_DIRECT	GO:0005634	Nucleus	38	KDM6A, STK11, SSFA2, ZNF652, IQGAP1, ZBTB38, CHD9, TOP1, NLRC4, ANK1, TPP2, BCL11B, USP10, INO80D, BAZ2A, AHNAK, HECTD1, DHX9, SLC25A6, ZFX, AFF3, SAMHD1, CDC27, SAFB2, ATRX, HES1, HDAC5, UBE2O, CYBA, ZMIZ2, MED16, PERM1, JAK1, RPL37A, SMC1A, SRCAP, KDM6B, TOB1	6.28E-05
UP_KEYWORDS	/	Isopeptide bond	15	SSFA2, ZBTB38, SAFB2, ATRX, TOP1, CHD9, SON, ZMIZ2, TPP2, BCL11B, EIF1AX, KHSRP, MAP4, BAZ2A, AHNAK	6.95E-05
GOTERM_CC_DIRECT	GO:0005737	Cytoplasm	36	BID, IL1R2, STK11, SSFA2, IQGAP1, CHD9, ANK1, TPP2, ASMTL, TIA1, USP10, NSMAF, BAZ2A, AHNAK, HECTD1, DHX9, TAOK1, DGKK, CD99, AFF3, EPRS, CDC27, PTPN12, FXR1, SAFB2, HES1, HDAC5, UBE2O, ACVR2B, FCGR2C, PERM1, CEMP1, JAK1, MAP4, SMC1A, TOB1	1.86E-04
GOTERM_MF_DIRECT	GO:0044822	Poly(A) RNA binding	14	DHX9, FXR1, SAFB2, TOP1, UBE2O, SON, EIF1AX, TIA1, KHSRP, MAP4, RPL37A, USP10, SMC1A, AHNAK	4.98E-04
UP_KEYWORDS	/	Chromatin regulator	7	ATRX, HDAC5, CHD9, KDM6A, SRCAP, BAZ2A, KDM6B	7.22E-04
UP_KEYWORDS	/	Nucleus	33	KDM6A, AGFG1, STK11, ZNF652, ZBTB38, CHD9, TOP1, TPP2, BCL11B, TIA1, USP10, BAZ2A, INO80D, AHNAK, DHX9, ZFX, SAMHD1, AFF3, CDC27, SAFB2, ATRX, HES1, HDAC5, UBE2O, SON, ZMIZ2, MED16, PERM1, KHSRP, SMC1A, SRCAP, KDM6B, TOB1	1.46E-03

### PPI Network Analysis of DEGs

**Figure 3:**
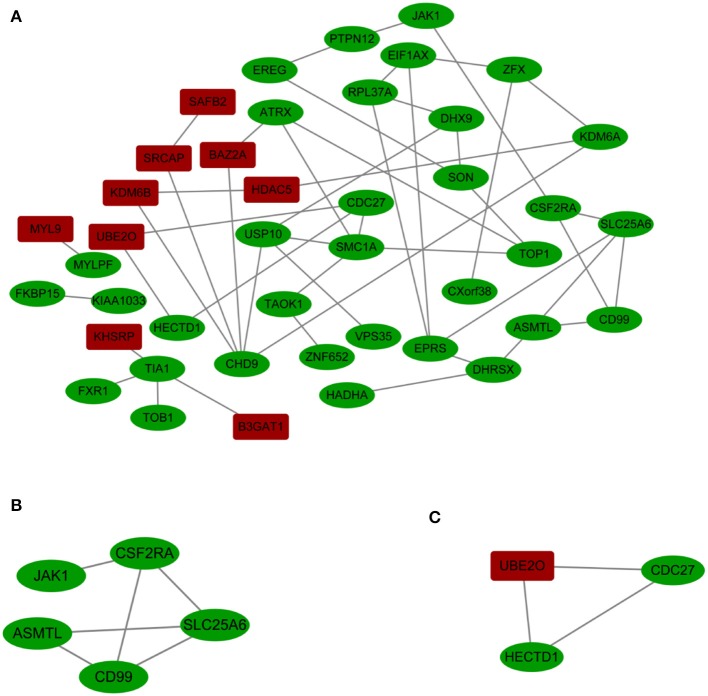
**(A)** Cytoscape network visualization of the 42 nodes and 49 edges that were obtained with interaction scores >0.4 according to the STRING online database. The nodes represent genes, and the edges represent links between genes. Red represents upregulated genes, and green represents downregulated genes. **(B,C)** Two key modules were identified by ClusterOne, which was used to identify network gene clustering.

A PPI network with 42 nodes and 49 edges was obtained; the network had an interaction score >0.4 according to the STRING online database (Figure 3A). The nodes correspond to genes, and the edges represent the links between genes. Red represents upregulated genes, and green represents downregulated genes.

We used ClusterOne in Cytoscape to perform network gene clustering to identify the key PPI network modules. As shown in Figures 3B,C, two key modules with one upregulated gene (*UBE2O*) and seven downregulated genes (*CDC27, HECTD1, JAK1, ASMTL, CD99, SLC25A6*, and *CSF2RA*) were identified. Furthermore, functional enrichment analysis indicated that these eight genes were mainly involved in protein binding, phosphoprotein, methylation, the nucleus and the cytoplasm (Table 3).

### Identification of Genes of Interest

We identified three downregulated X chromosome genes (*AP1S2, CSF2RA*, and *CD99*) that are involved in the immune system. PPI network analysis detected two key modules consisting of eight genes (*UBE2O, CDC27, HECTD1, JAK1, ASMTL, CD99, SLC25A6*, and *CSF2RA*). The tissue-specific gene expression analysis revealed that *CDC27* and *CD99* were specifically expressed in the hematologic/immune system. In addition, using the GeneCards database, we manually identified three additional genes of interest (*UBE2O, HECTD1*, and *CSF2RA*) that are potentially related to the pathogenesis of TS. Furthermore, we identified several other potentially involved genes among the genes with tissue-specific expression using the GeneCards database. All the genes of interest are shown in Table 4.

**Table 4:** Genes of interest.

**Up-regulated**			**Down-regulated**		
**Gene**	**Fold change**		**Gene**	**Fold change**	
	**Xm-XX**	**Xp-XX**		**Xm-XX**	**Xp-XX**
**HEMATOLOGIC/IMMUNE SYSTEM**					
UBE2O	2.549	2.801	CDC27	0.3711	0.3945
AGER	3.434	3.729	CD99	0.4601	0.4851
ANK1	5.540	2.630	HECTD1	0.4730	0.4768
			CSF2RA	0.3099	0.3510
			AP1S2	0.4263	0.3898
			DHX9	0.4569	0.4841
			EREG	0.2952	0.3238
			IL1R2	0.1627	0.3489
			PLXNC1	0.3487	0.3616
**CIRCULATORY SYSTEM**					
MYL9	10.411	9.513	MYLPF	0.2872	0.1968
**METABOLISM SYSTEM**					
IGFBP2	4.347	3.908			
B3GAT1	4.993	5.448			

## Discussion

In this study, we analyzed the DEGs in PBMCs from monosomy X TS patients and normal females. We performed independently for the 45,Xm and 45,Xp TS samples to narrow and strengthen the potential pathogenesis genes in TS. Several novel genes that had not been reported to be associated with this condition were identified by comparing Xm and Xp TS patients. This discovery-driven analysis included a genome-wide search, and these novel genes provide insight into the pathogenesis of TS.

TS is a common genetic condition caused by abnormal sex chromosomes where in the affected female individuals lose an entire copy or a portion of the X chromosome (17). The X chromosome contains many genes, and these genes are mainly involved in ovarian development and the immune and skeletal systems. Loss of the entire X chromosome or a portion of it can lead to haploinsufficiency of these genes, causing short stature (18, 19), gonadal insufficiency (20, 21), or maldevelopment of the lymphatic system (12, 22). In addition, cardiovascular abnormalities (23), metabolic syndrome (24) and intellectual disability (25) are also present in TS patients.

The XIST gene is involved in the X-inactivation process, which ensures gene dosage equivalence between males and females (26). The XIST gene is universally expressed in all cells; therefore, it can function as a positive control. In our study, the expression level of the XIST gene was lower in TS individuals than in normal individuals, which demonstrated the accuracy of this research method. We identified a total of 85 DEGs, consisting of 25 upregulated and 60 downregulated genes in monosomy X TS patients. But, the *SHOX* gene which is known to be involved in skeletal abnormalities (5) was down-regulated only in TS patients with paternally inherited X chromosome (Supplemental Table 1), probably due to its only moderate expression abundance in PMBC, was not identified as a being differentially expressed between 45,XO and 46,XX in our study. There existed limitations which may result in the lose of several feasible pathogenesis genes. But it is in line with the tissue-specific expression analysis, revealing that the most highly specific system in terms of the expression of the DEGs was the hematologic/immune system, which could explain the common occurrence of autoimmune diseases in TS patients. In TS patients, the main autoimmune diseases are autoimmune thyroiditis, type 1 diabetes and autoimmune enteritis. Among these disorders, the most common is autoimmune thyroiditis (13). Two possible mechanisms may explain the increased prevalence of autoimmune diseases in patients with TS. One plausible mechanism is that the X chromosome contains a large number of immune-related genes, and the altered X-linked gene dosage results in the loss of immune tolerance (27, 28). The other potential mechanism is that autoimmune diseases are induced by chromosome aneuploidy (22). Although the dataset is derived from PMBC, similar results were also observed in leukocyte RNA-expression profile and human fibroblast cell line, as well as by comparing amniotic fluid RNA expression of profile of Turner syndrome fetuses and female fetuses.

The PPI network and key module analyses identified two genes of interest (*CD99* and *CSF2RA*) that are involved in the hematologic/immune system. CD99, a cell surface glycoprotein, is encoded by the pseudoautosomal gene *MIC2* (29) and is involved in critical biological processes. In studies on CD99-deficiency in fetuses, Shin et al. (30) demonstrated that CD99 plays a key role in lymphocyte development. In addition, other studies have suggested that CD99 is important in peripheral immune responses and hematopoietic precursor cell differentiation (31). *CSF2RA* is a protein-coding gene that is located in the pseudoautosomal region of the X chromosome. The protein encoded by this gene is the alpha subunit of the heterodimeric receptor for colony-stimulating factor 2, a cytokine that controls the production, differentiation, and functions of granulocytes and macrophages. The data suggest that dysregulation of CD99 and CSF2RA might underlie the increased frequency of autoimmune diseases in females with TS.

Cardiovascular abnormalities, bicuspid aortic valve and associated aortic disease including coarctation of the aorta are common in TS (24). Our manual screening of tissue-specific gene expression identified one upregulated gene, *MYL9*, and one downregulated gene, *MYLPF*. The protein encoded by *MYL9* is the myosin light chain, which may regulate skeletal muscle contraction and is also associated with smooth muscle contraction (32). *MYLPF*, which was downregulated, encodes the regulatory light chain of striated muscle (33). These data suggest that both MYL9 and MYLPF may function in vascular muscles, and their dysregulation could play important roles in increasing the risk of aortic coarctation in females with TS.

Another characteristic of TS is short stature, and the *SHOX* gene is known to be involved in skeletal abnormalities (5). An upregulated gene, *IGFBP2*, encoding insulin-like growth factor binding protein 2, was identified in our trial. Insulin-like growth factor (IGF) regulates cartilage and bone development through the integrated action of IGF ligands, receptors and binding proteins (34, 35). Fisher et al. (36) found that overexpression of IGFBP2 inhibits IGF-mediated proliferation and reduces the proliferation of maturing chondrocytes and the formation of the periosteal bony collar, thus disrupting the balance of IGF/IGFBP2 activity and bone development. Further experiments should be performed to confirm the action of IGFBP2 in the pathogenesis of TS.

Genes involved in chromatin regulator were identified from our enriched GO terms analysis, including *ATRX, HDAC5, CHD9, KDM6A, SRCAP, BAZ2A*, and *KDM6B*. SRCAP, a SNF2-related chromatin-remodeling ATPase, was found to be elevated 4.563- and 6.930-folds in TS patients with Xm and Xp compared with normal female. SRCAP has various crucial roles in chromatin remodeling, gene expression, DNA damage response and cell division. Although lack of *in vivo* and/or *in vitro* experiments, by whole-exome sequencing, *SRCAP* was recently found to be the causative gene for Floating–Harbor syndrome (FHS), a rare human disease characterized by delayed bone mineralization and growth deficiency, mental retardation and skeletal and craniofacial abnormalities (37). *ATRX* (ATP-dependent helicase ATRX, X-linked helicase I), located in X, plays a key role in deposition of the histone variant H3.3 at telomeres and other genomic repeat, maintaining the heterochromatic modifications at these sites. Inherited mutations of the *ATRX* gene cause diverse changes in the pattern of DNA methylation, leading to an X-linked mental retardation (XLMR) syndrome most often accompanied by alpha-thalassemia syndrome. Trolle et al. (10) found genome wide hypomethylation with most differentially methylated positions in TS patients, suggesting the dysfunction of chromatin regulator. Further study need to be done illustrate whether the dosage of ATRX underlie the mechanism of TS.

In this study, we used discovery-driven analysis to identify DEGs and found five genes of interest (*CD99, CSF2RA, MYL9, MYLPF*, and *IGFBP2*) by constructing a PPI network and identifying key modules. In the future, more research should be conducted to study the relationship between these genes and TS.

## Data Availability Statement

The datasets generated for this study can be found in the GEO dataset: https://www.ncbi.nlm.nih.gov/geo/query/acc.cgi?acc=GSE46687, using code GSE46687.

## Author Contributions

HW performed the data analysis. HW and HZ contributed to the writing and revising of this manuscript. WZ, YX, NW, and BH sorted out the data. HS and JQ conceived and designed the experiments and revised the manuscript. All authors have seen and approved the final manuscript.

### Conflict of Interest

The authors declare that the research was conducted in the absence of any commercial or financial relationships that could be construed as a potential conflict of interest.
